# *Salmonella* Heterogeneously Expresses Flagellin during Colonization of Plants

**DOI:** 10.3390/microorganisms8060815

**Published:** 2020-05-29

**Authors:** Azhar A. Zarkani, Nieves López-Pagán, Maja Grimm, María Antonia Sánchez-Romero, Javier Ruiz-Albert, Carmen R. Beuzón, Adam Schikora

**Affiliations:** 1 Julius Kühn-Institut Federal Research Centre for Cultivated Plants (JKI), Institute for Epidemiology and Pathogen Diagnostics, Messeweg 11/12, 38104 Braunschweig, Germany; azhar.zarkani@julius-kuehn.de (A.A.Z.); maja.grimm@julius-kuehn.de (M.G.); 2 Department of Biotechnology, College of Science, University of Baghdad, 10071 Baghdad, Iraq; 3 Instituto de Hortofruticultura Subtropical y Mediterránea, Universidad de Málaga-Consejo Superior de Investigaciones Científicas (IHSM-UMA-CSIC), Dpto. Biología Celular, Genética y Fisiología, Campus de Teatinos, 29071 Malaga, Spain; nieves.lpg@uma.es (N.L.-P.); javieruizal@uma.es (J.R.-A.); cbeuzon@uma.es (C.R.B.); 4 Departamento de Genética, Facultad de Biología, Universidad de Sevilla, Apartado 1095, 41080 Seville, Spain; mtsanchez@us.es; 5 Current address: Departamento de Microbiología y Parasitología, Facultad de Farmacia, Universidad de Sevilla, Calle Profesor García González 2, 41012 Seville, Spain

**Keywords:** *Salmonella*, flagellin, colonization, crop plants, heterogeneous expression

## Abstract

Minimally processed or fresh fruits and vegetables are unfortunately linked to an increasing number of food-borne diseases, such as salmonellosis. One of the relevant virulence factors during the initial phases of the infection process is the bacterial flagellum. Although its function is well studied in animal systems, contradictory results have been published regarding its role during plant colonization. In this study, we tested the hypothesis that *Salmonella’s* flagellin plays a versatile function during the colonization of tomato plants. We have assessed the persistence in plant tissues of a *Salmonella enterica* wild type strain, and of a strain lacking the two flagellins, FljB and FliC. We detected no differences between these strains concerning their respective abilities to reach distal, non-inoculated parts of the plant. Analysis of flagellin expression inside the plant, at both the population and single cell levels, shows that the majority of bacteria down-regulate flagellin production, however, a small fraction of the population continues to express flagellin at a very high level inside the plant. This heterogeneous expression of flagellin might be an adaptive strategy to the plant environment. In summary, our study provides new insights on *Salmonella* adaption to the plant environment through the regulation of flagellin expression.

## 1. Introduction

The burden of human foodborne diseases is substantial. The most common illnesses resulting from unsafe food include diarrheal diseases, affecting 550 million people per year [1]. *Salmonella* is one of the four key global causes of diarrheal diseases. Indeed, the food-borne pathogen *Salmonella enterica* is associated with a number of diseases in a wide range of animal hosts. Therefore, *Salmonella*-related infections still represent an important health concern worldwide. Whether bacteria remain restricted to the gastrointestinal tract and local lymphatic system, or whether they spread to organs like the spleen or liver, is highly dependent on the individual host and the particular *Salmonella* strain. Severe cases of infection may lead to serious clinical problems, including death [2].

The flagellum is an important virulence factor, often involved in the initial phase of the infection. Flagella are long, helical appendages that allow bacteria to direct their movement towards nutrient sources or away from harmful substances [3,4]. *Salmonella enterica* serovar Typhimurium (*S.* Typhimurium) has approximately 6 to 10 peritrichous flagella distributed around the cell [5]. The flagellum is a complex structure made of approximately 30 different proteins that extends up to 20 µm beyond the cell surface [6]. Typically, it consists of three main segments: the basal body, the hook, and the filament. The basal body anchors the flagellum into the bacterial inner and outer membranes and includes rotor and stator protein complexes, necessary for force generation and flagellar rotation [7]. The flexible extracellular hook functions as a universal joint that connects the basal body with the third segment, the filament [8,9]. The filament forms a helical propeller and consists of approximately 20,000 subunits of flagellin. Many *Salmonella* serovars alternate flagellin expression between two antigenically different proteins, FljB and FliC, in a process known as flagellar phase variation. Usually only one of the flagellins is produced at a time in a given cell [5]. These flagellin variants display structural variability resulting in differences in swimming towards host cell surfaces. For example, bacteria expressing FliC-flagella are more efficient in the identification of target sites on host cell surfaces and therefore have an advantage when invading epithelial cells. They outcompete FljB-flagellated bacteria during intestinal colonization in both gastroenteritis and typhoid murine infection models even though their intracellular survival and the responses triggered on the host immune system are similar [10].

Besides being involved in bacterial motility and chemotaxis, flagella are also required for many other processes that contribute to a successful colonization of the host. In mammalian systems, physical forces between flagella and the host cell surface allow *Salmonella* to scan the host’s surface topology and to determine the optimal infection site [11]. Moreover, the flagellar filament seems to be important for intestinal cell adhesion, which enables triggering of membrane ruffling and initiate invasion [12].

While flagella actively contribute to the infection process during pathogenic interactions, flagellin may cause a disadvantage. Flagellin is one of the best-studied Pathogen-Associated Molecular Patterns (PAMPs) and is recognized in mammals and plants by the Toll-like receptor 5 (TLR5) and FLAGELLIN SENSING2 (FLS2) immune receptors, respectively [13,14,15]. Although PAMPs are considered to be conserved, several reports revealed that some bacteria have evolved divergent flagellin sequences, leading to reduced Pattern-Triggered Immunity (PTI) activation [16,17,18,19].

In plant systems, reports regarding the importance of flagella for the survival and colonization of *Salmonella* are conflicting. Shaw et al., reported that flagella do not have a role in attachment to tomato plants [20]. In contrast, other studies showed that the absence of a flagellar filament has an impact on adhesion to various plants. Berger et al., reported reduced adhesion to basil leaves for the *S.* Senftenberg *ΔfliC* mutant [21], whereas in *Arabidopsis thaliana* the *ΔfliC* mutant exhibited enhanced colonization of roots [22]. Differences in the role of flagella in colonization of rhizosphere versus phyllosphere have been recently suggested [23]. These authors reported decreased adhesion levels to corn salad (*Valerianella locusta*) leaves for strains lacking flagella filaments. Hence, flagella were proposed to play an important role during both adhesion and motility, while in contact with corn salad. This discrepancy in results might be attributed to the different plant models used in each study. Additionally, variations in the physiological conditions of the plants used in the different studies, as well as analogous variations in the corresponding *Salmonella* strains, could have added to those discrepancies. Noteworthy, a recent study reported differences in the structures and motility functions of FliC and FljB under high-viscosity conditions. The authors stated that *Salmonella* strain expressing FljB showed a higher motility than the one expressing FliC under high viscosity. They attributed these differences to the density of D3 domain, which was much lower in FljB than FliC, offering more flexibility and mobility than to that of FliC [24].

In this work, we hypothesize that flagella might play a versatile role during colonization of plants. We used tomato (*Solanum lycopersicum* cultivar Moneymaker) as a representative crop plant, which fruits are widely consumed and have been associated to several salmonellosis outbreaks [25]. We assessed the ability of *S.* Typhimurium wild type strain 14028s and a double flagellin mutant *S.* Typhimurium *ΔfljBΔfliC* to persist inside the plant, as well as to reach distal, non-inoculated tissues of the plant. We compared the respective abilities of these strains using competitive index assays. The defense response of tomato plants to the presence of wild type *Salmonella* strains and strains lacking flagella was evaluated through gene expression analysis of marker genes. We also analyzed the expression of motility and adhesion-related bacterial genes in response to plant media. Finally, we evaluated the expression of *fliC* at the single-cell level following expression of a chromosome-located transcriptional fusion to Green Fluorescent Protein (GFP) by confocal microscopy and flow cytometry. We analyzed *fliC* expression in bacteria growing in medium supplemented with plant extract, and also in bacteria directly extracted from the plant apoplast. Our results show that the majority of cells within the *S.* Typhimurium apoplastic population down regulate the expression of flagella. However, a small subpopulation maintains flagellar expression *in planta* at a very high level. Heterogeneous expression of flagella might be an adaptive strategy of *Salmonella* to efficiently colonize the plant environment: non-motile cells not expressing flagellin could reduce recognition by the plant immune system or display increased fitness, while those retaining motility might be ready to colonize new ecological niches such as, for example, the herbivore. Taken together, our results provide new insights on how *Salmonella* adapts to the plant environment and on which strategies *Salmonella* uses in order to persist in this environment.

## 2. Materials and Methods

### 2.1. Bacterial Strains, Culture Conditions and Media Preparation

*Salmonella enterica* serovar Typhimurium strain 14028s (*S*. Typhimurium 14028s), resistant to rifampicin, was used in this study as the wild type reference strain. In addition, a flagellin double mutant *Salmonella enterica* serovar Typhimurium 14028s *∆fljB∆fliC*, (*S.* Typhimurium *∆fljB∆fliC*) kindly provided by Michael Hensel (University of Osnabrück, Osnabrück, Germany) was used. Bacteria were grown in Luria-Bertani (LB) broth (Carl Roth GmbH & Co., KG, Karlsruhe, Germany) or on LB agar plates. Plant-based media, namely lettuce medium (LM) and tomato medium (TM), were prepared as described previously by [26,27], respectively. Minimal medium (MM) was used as a control medium and was prepared as described by [27]. Xylose-lysine-desoxycholate (XLD) agar (Carl Roth GmbH & Co., KG) was used as a selective medium for *Salmonella*. Antibiotics were used at the following concentrations: rifampicin 50 mg/L for *S*. Typhimurium 14028s, rifampicin 50 mg/L plus kanamycin 50 mg/L for *∆fljB∆fliC*, and chloramphenicol 50 mg/L for *fliC-gfp* strain. Bacteria were incubated overnight on LB and XLD agar plates at 28–37 °C, or at 25–28 °C on plant-based media. All strains used in this study are listed in Supplementary Table S1.

### 2.2. Fluorescent Labeling of Salmonella Strains

In order to visualize colonization patterns, *S*. Typhimurium 14028s and *S*. Typhimurium *∆fljB∆fliC* were GFP-labeled with the pSM1890 GFP plasmid [28]. The preparation was performed according to [29]. Briefly, *Salmonella* strains were allowed to mate with *E. coli* carrying the IncQ plasmid pSM1890 GFP (derived from the IncQ plasmid pIE723 [30] and *E. coli* carrying the helper plasmid R751 [31]. Cells were re-suspended from LB plates into 1 mL of a 10 mM MgCl_2_ solution. The cell suspensions were combined, mixed and centrifuged and the supernatant discarded. The pellets were re-suspended in the remaining supernatant. The solution was placed on a filter disc (0.22 µm, Durapore membrane filters, Merck, Darmstadt, Germany) on LB agar and incubated overnight at 28 °C. Cells were re-suspended by vortexing the filter in 5 mL of 10 mM MgCl_2_ solution and transconjugants were selected by plating serial dilutions on LB. After overnight incubation at 28 °C, green fluorescent colonies were picked and plated on XLD agar for further detection on the following day.

In addition, a transcriptional fusion to the *gfp* gene was generated in *S*. Typhimurium 14028s downstream of the stop codon of the *fliC* ORF. The source of the promoterless *gfp* ORF including its ribosomal-binding site, and the chloramphenicol resistance cassette, used for selecting the integration by allelic exchange of the *gfp*-containing DNA fragment, was pZEP07 [32]. The construct was integrated into the chromosome of *S. enterica* using the Lambda Red recombination system [33].

### 2.3. Plant Cultivation

Tomato (*Solanum lycopersicum* cultivar Moneymaker) seeds were surface sterilized with 70% ethanol for 1 min followed by incubation for 3 min in 3% sodium hypochlorite (NaClO) solution. The seeds were later vigorously washed with sterile distilled water. Seeds were germinated for 7–10 days in Petri dishes on sterile ¼-strength Murashige and Skoog (MS) agar medium (Sigma-Aldrich Chemie GmbH, München, Germany), pH 5.4 including vitamins and 5 g/ L sucrose. Seedlings were grown under sterile conditions with a light intensity of 150 µmol m^2^/s (16 h photoperiod) at 22 °C for either two additional weeks in sterile glass pots (for spray inoculation), or for 2 days in 50 mL conical tubes, containing 20 mL ¼ MS liquid medium for microscopic analysis.

In order to evaluate the survival of the bacteria in leaves, plants were grown under greenhouse conditions in standard bedding substrate (substrate 1, Klasmann-Deilmann GmbH, Geeste, Germany) at 22 °C and 16 h photoperiod for 4–5 weeks. The plants were watered as needed from the bottom to avoid contamination of the non-inoculated leaf parts.

### 2.4. Confocal Laser Scanning Microscopy (CLSM)

Tomato plants were grown under sterile conditions in order to visualize the colonization patterns of *Salmonella.* Seven to ten-day old plants were transferred to conical tubes containing ¼-strength MS liquid medium. Plants were allowed to adapt to the medium for 24 h before the medium was inoculated with a final OD_600 nm_ = 0.1 corresponding to 10^8^ colony forming units/mL (CFU/mL) of either *S*. Typhimurium 14028s-GFP or *S*. Typhimurium *∆fljB∆fliC*-GFP strain. Plants were sampled (leaves and roots) 24 h post inoculation (hpi), stained with propidium iodide (PI) solution (1 µg/ mL) for 5 min and mounted on a microscope slide in 4′, 6-diamidine-2′-phenylindole dihydrochloride (DAPI) solution (10 µg/ mL). Confocal laser scanning microscopy was performed using SP8 microscope (Leica Microsystems, Wetzlar, Germany) with excitation 405 nm, emission 430–480 nm (blue), excitation 488 nm, emission 500–550 nm (green), excitation 561 nm, emission 600–680 nm (red) including autofluorescence of chloroplasts.

### 2.5. Persistence of Salmonella in Plants

To assess the survival of *Salmonella* inside tomato leaves, plants were grown in substrate under greenhouse conditions as described above. Leaves were inoculated with *S*. Typhimurium 14028s and *S*. Typhimurium *∆fljB∆fliC* strains using either syringe infiltration or dip-inoculation. To avoid contamination of the entire plant, the non-inoculated parts were covered with plastic bags until the inoculated parts dried (Supplementary Figure S1). Bacterial inocula were prepared using fresh bacterial colonies grown on LB plates. Biomass thus obtained was adjusted to 10^7^ CFU/mL (infiltration) or 10^8^ CFU/mL (dipping) using 10 mM MgCl_2_. Additionally, some plants were either inoculated with 10 mM MgCl_2_ or non-treated (N.T.) and used as controls. Leaves were sampled three hours (0 day), 7 and 14 days post inoculation (dpi). Five mm diameter leaf discs were obtained using a sterile biopsy punch (GlaxoSmithKline PLC, Brentford, UK) and homogenized in 1 mL 10 mM MgCl_2_ using a tissue homogenizer (Xenox, Götze, Berlin, Germany). Serial dilutions were prepared in duplicates and 10 µl of each dilution was dropped in duplicates onto XLD agar plates. CFUs were counted after an overnight incubation at 37 °C. The experiment was performed in three (infiltration) or four (dipping) replicates (one leaf per plant and replicate). Student’s *t*-test was applied and *p* ≤ 0.05 was considered significant. No colonies were found in negative control (N.T.) or MgCl_2_-treated plants.

### 2.6. Translocation of Salmonella within Tomato Plants

To test the requirement of flagella in systemic colonization of tomato, plants were grown under greenhouse conditions as described previously. Leaves were inoculated with *S*. Typhimurium 14028s or *S*. Typhimurium *∆fljB∆fliC* strains following the methods described above (infiltration or dipping). Leaves (inoculated and non-inoculated) were sampled 5 h (0 dpi), 7 and 14 dpi, cut with sterile scissors and placed in 50 mL conical tubes containing 10 mL Buffered Peptone Water (BPW) (Carl Roth GmbH & Co. KG). Tubes were incubated at 37 °C overnight under shaking conditions (140 rpm) and 10 µL of the suspension was transferred to 190 µL Rappaport Vassiliadis Broth (RVS) (Carl Roth GmbH & Co. KG) and incubated overnight at 42 °C. Ten µL of both enrichments (BPW and RVS) was dropped on XLD agar plates to confirm the presence of *Salmonella* after overnight incubation at 37 °C. The experiment was conducted in seven (infiltration) or eight (dipping) replicates. One leaf per plant per treatment and replicate was used.

### 2.7. Competitive Bacterial Colonization Assays

Competitive index (CI) assays were carried out as previously described in [34]. Briefly, leaves from four to five-week old tomato plants, growing under greenhouse conditions, were infiltrated with a 5 × 10^5^ CFU/mL of a mixed bacterial suspension, containing equal CFU of wild type (*S*. Typhimurium 14028s) and mutant derivative strain (*S*. Typhimurium *∆fljB∆fliC*), using a blunt syringe. Serial dilutions of the inoculum were plated onto LB agar and LB agar with kanamycin to confirm dose and relative proportion between the strains, which should be close to one. Seven- or 14-days post-inoculation (dpi), five 10 mm-diameter leaf discs were taken from the infiltrated area of each leaf and homogenized together by mechanical disruption into 1 mL of 10 mM MgCl_2_. Then, bacteria were enumerated by plating serial dilutions onto LB agar supplemented with cycloheximide, and the colonies obtained were replica-plated onto LB agar and LB agar with kanamycin, to differentiate the strains within the mixed infection. Bacterial enumeration was carried out in the dilution displaying between 50 and 500 colonies per plate. The CI is defined as the mutant-to-wild type ratio within the output sample divided by the mutant-to-wild type ratio within the input (inoculum), which should be close to one [35,36]. CIs presented were obtained from six plant replicates. Mean CI values are shown. Error bars represent standard error. Each CI was analyzed using a homoscedastic and 2-tailed Student’s *t*-test and the null hypothesis that mean index is not significantly different from 1 (*p* < 0.05).

### 2.8. Quantitative Real Time PCR Analysis

To assess the response of tomato to the presence of *Salmonella* and to evaluate the role of flagella in this response, *S*. Typhimurium 14028s and *S*. Typhimurium *∆fljB∆fliC* were sprayed onto 3-week old sterile plants. Plants were grown on ¼-strength MS agar medium. In order to allow the bacteria to adapt to the plant environment, bacteria were pre-grown overnight on tomato-based medium (TM) at 25–28 °C. Plants were spray-inoculated with OD_600 nm_ = 0.1 (10^8^ CFU/mL). Leaves were sampled at 0, 6 and 24 hpi and samples were pooled from three plants per treatment. About 100 mg of leaf tissue was homogenized in a TissueLyser (Qiagen, Hilden, Germany) and the total RNA was extracted using peqGOLD TriFast Reagent (Peqlab, Darmstadt, Germany) according to the manufacturer protocol. DNase treatments, using PerfeCTa DNase I, and cDNA synthesis were performed using 1 µg of total RNA and qScript cDNA Synthesis kit (Quanta BioSciences, Gaithersburg, MD, USA). Target cDNA was then amplified in a 20 µL reaction mixture containing 5 µL of sample DNA and LUNA Master Mix (New England Biolabs, Frankfurt, Germany) according to the manufacturer procedure. Reactions were run for 5 min at 95 °C, followed by 40 cycles of 30 s at 95 °C, 30 s at 58 °C and 60 s at 72 °C in the CFX connect System (Bio-Rad, München, Germany). Primers used for the qPCR are listed in Supplementary Table S2. Relative gene expression was normalized to the expression of the *Actin* gene. The experiment was performed in 6 replicates. Tukey HSD Test was applied with 95% family-wise confidence level on R version 3.6.1 and *p* ≤ 0.05 was considered significant (Supplementary Table S3). Additionally, the experiment was repeated using 100 nM flg22 peptide (*QRLSTGSRINSAKDDAAGLQIA)* (AnaSpec. Inc., Fremont, CA, USA), as a control. The samples were obtained at 0 and 6 hpi and the experiment performed in 3 replicates.

### 2.9. Salmonella Response to Plant Media

Two experimental approaches were designed in order to test how well *Salmonella* adapts to the plant host and uses potential nutrients available in the plant environment. The first approach was intended to test the ability of the plant media to support growth of *Salmonella*, hence *S*. Typhimurium 14028s and *S*. Typhimurium *∆fljB∆fliC* strains were grown in liquid tomato-based medium (TM) overnight at 25–28 °C. The initial bacterial concentration was set to OD_600 nm_ = 0.01 and samples were taken at different time points. Serial dilutions were prepared in duplicates and 10 µl of each dilution was dropped onto XLD agar plates, and CFUs were quantified after overnight incubation at 37 °C. The experiment was performed in three replicates.

The second experimental approach was designed to assess the expression of *fljB, fliC* and *fimA* in *Salmonella* in response to different media. *S*. Typhimurium 14028s and *S*. Typhimurium *∆fljB∆fliC* were cultured in LB liquid medium overnight at 37 °C with aeration (150 rpm). Cells were pelleted at low speed (1500× *g*, 10 min), washed and re-suspended in 1 mL of 10 mM MgCl_2_. Two mL of the suspension (10^8^ CFU/mL) was pipetted into cellulose ester dialysis membrane tubes with a pore size of 100 kD (Spectrum Europe, Breda, The Netherlands), which were knotted at both ends using dental floss. The dialysis membranes were placed into 50 mL conical tubes containing 30 mL of TM, LM, LB (positive control), or MM (negative control) media. The conical tubes were incubated at 25–28 °C for 24 h while shaking at 130 rpm. Subsequently, 2 × 0.5 mL from each dialysis membrane were mixed with RNAprotect (Qiagen), incubated for 5 min at room temperature and centrifuged at high speed (4000× *g*, 10 min). Total RNA was extracted from the bacterial samples using the RNeasy Mini Kit (Qiagen) according to the manufacturer instructions. DNase digestion and cDNA synthesis were performed using Maxima H Minus First Strand cDNA Synthesis Kit (ThermoFisher Scientific, Braunschweig, Germany). The qPCR analysis was performed as described above. Primers used for the qPCR are listed in Supplementary Table S2. Relative gene expression was normalized to the expression of the *rfaH* gene from *Salmonella.* All treatments were performed in five replicates. Tukey HSD Test was applied with 95% family-wise confidence level on R version 3.6.1 and *p* ≤ 0.05 was considered significant (Supplementary Table S3).

### 2.10. Western Blot Analysis

*S*. Typhimurium 14028s and *S*. Typhimurium *∆fljB∆fliC* were cultured in dialysis membranes merged into conical tubes containing different media, as described earlier. Half mL of the bacterial culture was sampled from the dialysis membranes and centrifuged at low speed (1500× *g*, 10 min, 4 °C) in order to reduce shearing and loss of flagella. Pellets were re-suspended in 100 µL of 10 mM MgCl_2_. Proteins were extracted using methanol/chloroform. Protein concentration was measured at OD_595 nm_ using Bradford assay Roti^®^ Quant 5 x concentrated (Carl Roth GmbH & Co. KG). Five µg proteins were separated on SDS-PAGE using 12% polyacrylamide gels and electrophoretically transferred to a PVDF membrane (Immun-Blot^®^ PVDF, Bio-Rad) for the following western blot analysis. Anti-flagellin FliC monoclonal antibody (1:5000; InvivoGen, San Diego, CA, USA) was used as a primary antibody and goat anti-mouse polyclonal-HRP (1:5000; Carl Roth GmbH & Co. KG) was used as a secondary antibody. Bands were detected after adding substrate mixture (SERVA Electrophoresis GmbH, Heidelberg, Germany) using an Optimax X-ray film processor (PROTEC Medizintechnik GmbH & Co., Oberstenfeld, Germany). The experiment was performed in three replicates.

### 2.11. Quantification of Flagellin Protein in Different Media

*Salmonella* strains were grown and proteins were extracted as described above. We used 2 × 50 µL of each protein sample to coat clear bottom and high binding 96-well plates (Greiner bio-one GmbH, Frickenhausen, Germany). Coating was done for 3 h at 37 °C, the 96-well plates were subsequently washed and proteins were blocked overnight using 10% milk in PBS. Afterwards, samples were incubated with anti-flagellin FliC monoclonal antibody (1:1000; InvivoGen) for 1 h followed by another 1 h incubation with goat anti-mouse polyclonal-HRP (1:5000; Carl Roth GmbH & Co. KG). After each incubation step, 4 washing steps were performed. To measure absorbance, TMB substrate kit (ThermoFisher Scientific) was added and incubated in darkness for 30 min followed by addition of the stop solution (ThermoFisher Scientific). Measurements were carried out using TriStar^2^S multimode Reader (Berthold Technologies GmbH & Co., Wildbad, Germany) at 450 nm and 652 nm wavelengths applying a single endpoint protocol. To calculate the percentages of flagellin present, the values from the 652 nm measurement were subtracted from those obtained at 450 nm in order to correct the optical imperfections in the microplate. B0 (No antigen was added) and NSB (No primary antibody was added) were used as controls for non-specific binding between primary-secondary antibodies and antigen-secondary antibody, respectively. The experiment was performed in 3 replicates.

### 2.12. Flow Cytometry and Confocal Laser Scanning Microscopy (CLSM)

*Salmonella* cells from steady-state cultures in LB were washed with 10 mM MgCl_2_ and diluted in TM or LB at OD_600_ = 0.1, followed by incubation at 28 °C with shaking (aerophilia) or without shaking (microaerophilia) for time-course flow cytometry analyses. At different time points 300 µL of each culture were washed with 10 mM MgCl_2_ and analyzed by flow cytometry. For flow cytometry analysis of apoplast-extracted bacteria, tomato leaves were syringe infiltrated at OD_600_ = 0.1 and both inoculum and apoplast-extracted bacteria were used. To recover bacteria from tomato leaves an apoplastic fluid extraction was carried out at the indicated time point post inoculation as previously described by [34]. Summarily, the apoplastic fluid was obtained by pressure infiltrating a whole leaf with 10 mL of 10 mM MgCl_2_ solution inside a 20 mL syringe. Following five cycles of pressure application, the flow-through was removed and placed in a fresh 50 mL tube. Both tubes were centrifuged for 30 min at low speed (900× *g*) at 4 °C. Pellets were re-suspended into 1 mL MgCl_2_ and analyzed by flow cytometry. Cultures and apoplast-extracted bacterial suspensions were collected using a BD FACSVerse cytometer and data were analyzed with Kaluza Analysis Software (Beckman Coulter, Brea, CA, USA). To analyze cytometry data the following conventions were applied: all SSC, FSC, and fluorescence zero values were excluded. Data were excluded that fell within the forward scatter (FSC) and side scatter (SSC) region where significant counts appeared in “buffer only” controls. FSC and SSC medians were calculated and a series of circular gates expanding out from the FSC and SSC medians were applied. All data were collected for 100,000 events per sample and were compared with the data from the control strain (without GFP reporter) to establish the fraction of fliC^ON^ cells. Live/dead staining was carried out using propidium iodide at 20 mM (Sigma, St. Louis, MO, USA). Each independent experiment included two replicate samples, as indicated for each figure. Figures show typical results.

For microscopy images of apoplast-extracted bacteria, samples were stained with 20 µM of FM4-64 (*N*-3-triethylammoniumpropyl-4-6-4-diethylaminophenylhexatrienylpyridinium dibromide) (Thermo Fisher Scientific, Waltham, MA, USA) and observed under either a SP8 microscope (Leica Microsystems) or a confocal microscope LSM 800 (Zeiss, Wetzlar, Germany) with excitation 488 nm, emission 500–533 nm for GFP and excitation 488 nm, emission 604–674 nm for FM4-64.

## 3. Results

### 3.1. Salmonella Does Not Require Flagella to Persist and to Colonize Plants

**Figure 1:**
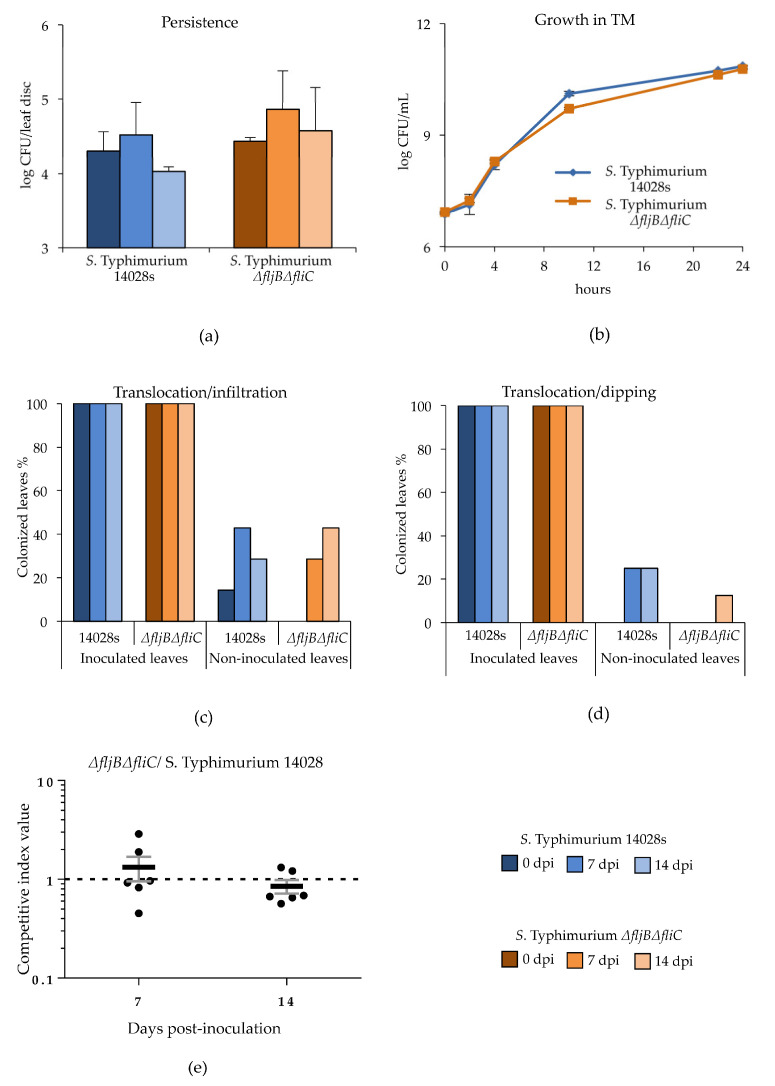
*Salmonella* persistence in and on leaves does not require flagella. *Salmonella enterica* serovar Typhimurium strain 14028s (*S*. Typhimurium 14028s) and the double *S*. Typhimurium *ΔfljBΔfliC* mutant were infiltrated at OD_600 nm_ = 0.01 (ca. 10^7^ CFU/mL) into tomato leaves. The presence of viable colony forming units (CFUs) was assessed during the following 14 days in cutout leaf discs. Both *Salmonella* strains persisted at the same level after infiltration (**a**). To monitor *Salmonella* growth in tomato-based (TM) medium, the number of CFU of *S*. Typhimurium 14028s and the double mutant *S*. Typhimurium *ΔfljBΔfliC* was assessed during 24 h post inoculation (hpi) into TM (**b**). Both strains reached the steady state during the 24 h, no difference between the wild type and the mutant proliferation rates was observed. Translocation of bacteria to non-inoculated leaves was verified using two inoculation techniques: infiltration (**c**) and dipping (**d**). After inoculation of leaves with bacterial suspensions (10^7^ CFU/mL for infiltration or 10^8^ CFU/mL for dipping), leaves were cut at the indicated days post inoculation (dpi) and subsequently incubated in enrichment media Buffered Peptone Water (BPW) and Rappaport Vassiliadis Broth (RVS). Graphs represent the translocation efficiency after infiltration (**c**) or dipping (**d**) inoculation. The enrichment cultures in RVS medium were plated on XLD-agar medium. The bars represent the percentage of plants for which non-inoculated leaves were positive for *Salmonella* from an average of seven (infiltration) and eight (dipping) replicates (one leaf per plant per treatment). (**e**) Direct comparison between persistence of *S*. Typhimurium 14028s and the double mutant *S*. Typhimurium *ΔfljBΔfliC* was assessed using competitive index (CI) assays. Both strains were co-inoculated by infiltrating a 5 × 10^5^ CFU/mL with a 1:1 proportion of these two strains. Bacteria were extracted 7 and 14 dpi from tomato leaves and plated on LB plates for CFU determination. Replica plating was carried out in LB and LB plus kanamycin to differentiate between the two strains. CIs presented are representative results from six replicates. Mean CI values are shown. Errors bars represent standard error. Each CI was analyzed using a homoscedastic and 2-tailed Student’s *t*-test and the null hypothesis that mean index is not significantly different from 1, *p* < 0.05 were considered significant.

In order to verify how flagellin, and therefore the ability to form a functional flagella, influences the persistence of *Salmonella* in plants, we analyzed the persistence of the wild type *Salmonella enterica* serovar Typhimurium strain 14028s (*S*. Typhimurium 14028s) and the double mutant *S*. Typhimurium *ΔfljBΔfliC* in tomato plants (*Solanum lycopersicum* cultivar Moneymaker). The number of colony forming units (CFU) within inoculated tissues was assessed 14 days post infiltration (dpi). We observed no differences in the number of CFU recovered from leaves inoculated with the *S*. Typhimurium *ΔfljBΔfliC* mutant strain *versus* those from leaves inoculated with the wild type *S*. Typhimurium 14028s (Figure 1a). These results are in line with our previous reports of *Salmonella* persistence in tomato plants [27], and indicate that the presence of functional flagella is not required for persistence of *Salmonella* in plants. Growth of both strains was also very similar in tomato-based medium (TM) (Figure 1b) [27].

Next, we asked whether flagella are required for systemic colonization of tomato plants. To this end, we monitored the presence of the wild type *S*. Typhimurium 14028s and the double *S*. Typhimurium *ΔfljBΔfliC* mutant in distal, non-inoculated leaves of previously inoculated plants, following either infiltration or dipping as inoculation methods (Supplementary Figure S1). Remarkably, cells of both *Salmonella* strains were found in distal non-inoculated parts of the plant. The highest percentage of leaves that tested positive for *Salmonella* was observed after infiltration, reaching a maximum of 40% at 7 dpi (Figure 1c).

Although the percentage of non-inoculated leaves that tested positive were lower after dipping, 20% of the leaves inoculated with *S*. Typhimurium 14028s at 7 and 14 dpi, and 10% of those inoculated with the *S*. Typhimurium *ΔfljBΔfliC* mutant were still colonized, respectively (Figure 1d). Importantly, the presence of *Salmonella* in the plant apoplast caused no observable disease symptoms, even 14 days after infiltration (Supplementary Figure S2).

These results encouraged us to test whether the wild type strain had any advantage over the *S*. Typhimurium *ΔfljBΔfliC* mutant during persistence in tomato plants, using mixed infections and calculating the corresponding competitive indices (CIs). Competitive assays are very sensitive and can accurately measure small differences in bacterial performance within the host, such as in host colonization. Those assays have been optimized for *Salmonella* in animals [37], and in plants systems for several bacterial pathogens [38,39]. Equal amounts of *S*. Typhimurium 14028s and the *S*. Typhimurium *ΔfljBΔfliC* mutant were co-inoculated by infiltration into tomato leaves at a final concentration of 5 × 10^5^ CFU/mL. This inoculation dose has been shown before to prevent trans-complementation between virulent and attenuated derivatives of plant pathogenic *Pseudomonas syringae*. In this experimental setting, co-inoculated strains have been shown to grow as they would in individual infections [38]. Since the competitive index (CI) is calculated as the test strain (mutant in this case)-to-wild type output ratio divided by their input ratio, a CI significantly smaller than one indicates a growth defect for the mutant strain being tested, and a CI significantly higher than one would indicate a competitive advantage for the mutant. The CI calculated after infiltration of *S*. Typhimurium *ΔfljBΔfliC* mutant in mixed infection with the wild type strain was not significantly different from 1.0 neither 7 nor 14 dpi (Figure 1e), suggesting that the ability to persist and to colonize plants in *Salmonella* does not rely on the function of flagella or the expression of flagellin. Analysis of a single *S*. Typhimurium *ΔfliC* mutant strain versus the *S*. Typhimurium 14028s wild type using CI in the same experimental conditions and time points rendered the same results (Supplementary Figure S3).

### 3.2. Salmonella Strain Lacking Flagellin Displays a Colonization Pattern on Tomato Leaves Similar to That of the Wild Type

**Figure 2:**
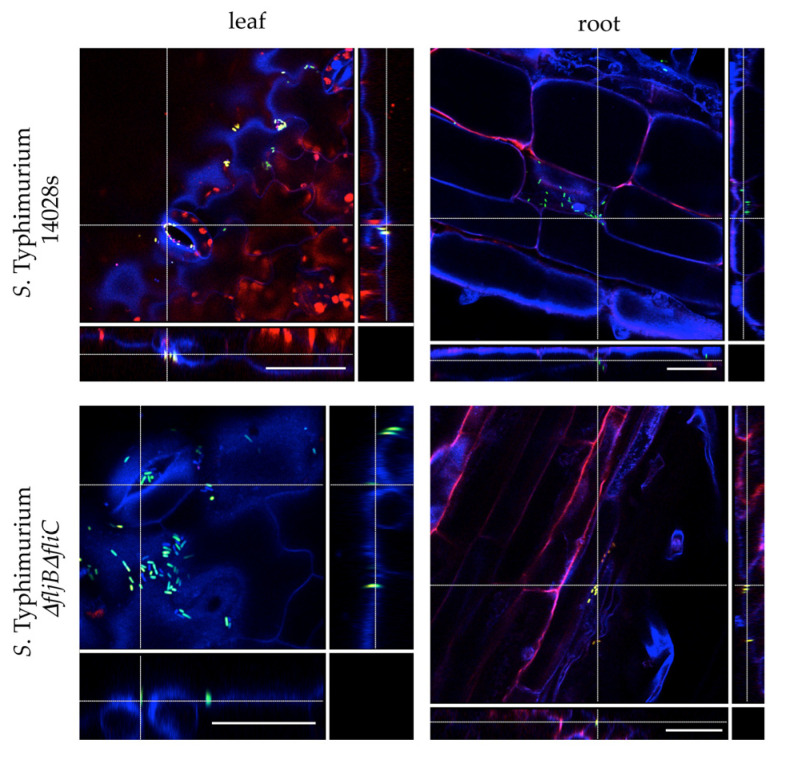
Attachment to plants does not require flagella. *Salmonella enterica* serovar Typhimurium strain 14028s (*S.* Typhimurium 14028s) and the double mutant *S*. Typhimurium *ΔfljBΔfliC* were labeled with the green fluorescent protein (GFP) using the pSM1890 GFP-plasmid. Plants were dip-inoculated with a 10^8^ CFU/mL suspension of GFP-expressing *Salmonella*. Attachment of *Salmonella* to plant roots and leaves was assessed after 24 h, using the confocal laser scanning microscopy with the following set up: excitation 405 nm, emission 430–480 nm (blue); excitation 488 nm, emission 500–550 nm (green) and excitation 561nm, emission 600–680 nm (red). Auto-fluorescence of chloroplasts is indicated in red and GFP-labeled *Salmonella* cells are indicated in green. Scale bars indicate 20 μm. The cross lines in orthogonal scaling are pointing the corresponding areas.

Next, we wondered if the colonization pattern on tomato plants is altered when *Salmonella* is deprived of flagella. Wild type *S*. Typhimurium 14028s and *S*. Typhimurium *ΔfljBΔfliC* double mutant were labeled with the green fluorescent protein (GFP) and plants were submerged into ¼-strenght MS medium and inoculated with GFP-expressing bacteria. Colonization patterns were assessed 24 h post inoculation (hpi) using confocal laser scanning microscopy (CLSM), evaluating both roots and leaves as potential entry sites. We observed no differences between the colonization patterns of wild type and mutant strains. Both strains assosiated to structures akin to root cells (Figure 2) and colonized the epidermis of tomato leaves. Similar to wild type bacteria, double mutant bacteria were observed gathering around stomata openings (Figure 2).

### 3.3. Expression of Flagellin-Coding Genes Is Down-Regulated in Plant-Mimicking Media

**Figure 3:**
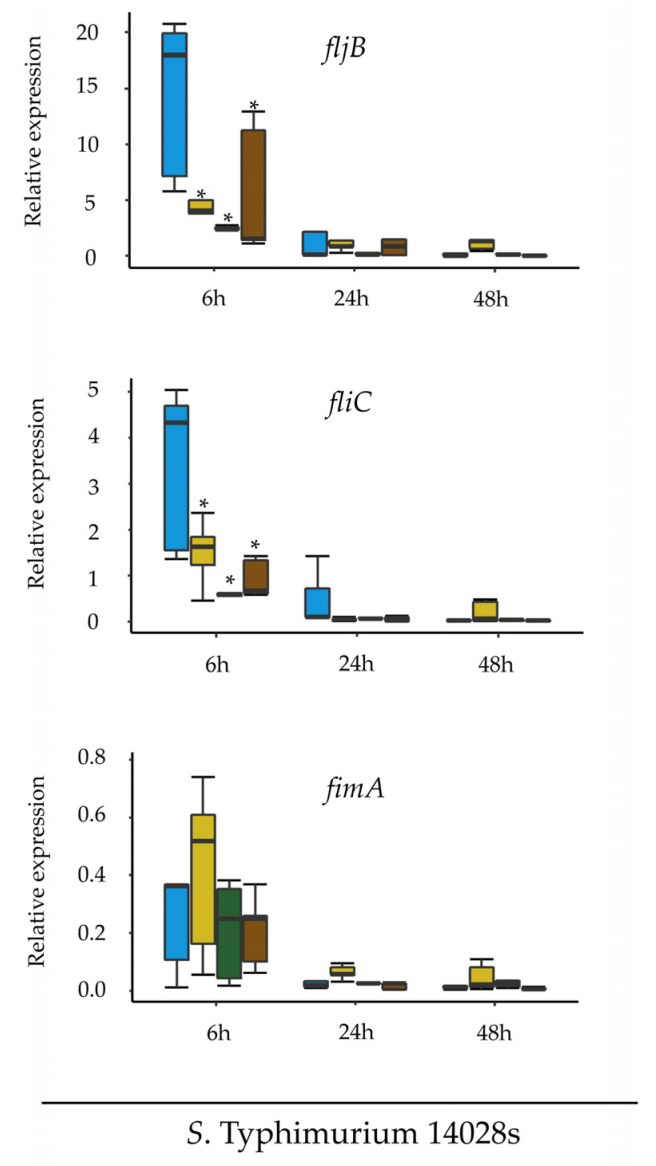

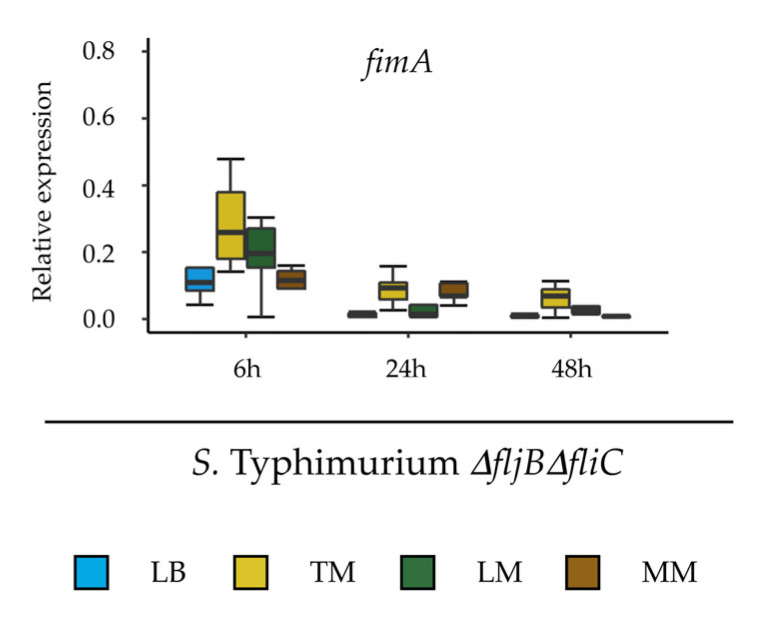
Flagellin expression is reduced in plant-based media. Expression of the flagellin *fljB*, *fliC* and the adhesin *fimA* genes was assessed 6, 24 and 48 h after inoculation of *S.* Typhimurium 14028s into plant-based (LM or TM) media. Inoculations into LB and minimal media (MM) were used as controls. The expression of *fimA* was assessed additionally in the *ΔfljBΔfliC* mutant. The amount of mRNA was normalized to the expression level of *Salmonella rfaH*. Asterisks indicate *p* ≤ 0.05 in Tukey HSD test. For more information about significant differences among treatments, please see Supplementary Table S3.

One possible explanation for flagellin not having an impact on *Salmonella* persistence or plant colonization would be that flagellin expression is down-regulated after bacterial entry into the plant tissue. To evaluate this possibility, expression of flagellin-coding *fljB* and *fliC* genes was determined first in media supplemented with plant extracts. Expression levels for these two genes were assessed at three different time points during bacterial replication in tomato-based medium (TM) [27] and, to evaluate a potential influence of diverse plant environments, expression was also assessed in lettuce-originated medium (LM) [29], as well as in a minimal medium (MM) with similar composition to TM and LM media but without added plant extracts. LB medium was used as a reference. All media were inoculated with equal amounts of wild type *S*. Typhimurium 14028s and cells were harvested 6, 24 and 48 h thereafter. The expression of *fljB* and *fliC* was reduced in both plant-supplemented media as soon as 6 hpi (Figure 3).

The *fimA* gene encoding for type 1 fimbriae (major subunit) was used as a control. Fimbriae play a role in mediating *Salmonella* adherence to eukaryotic cells [40]. In contrast, expression levels of the control gene *fimA* at 6 hpi were very similar in all media for both strains. At later time points, expression of *fljB*, *fliC* and *fimA* genes was significantly reduced (Figure 3).

**Figure 4:**
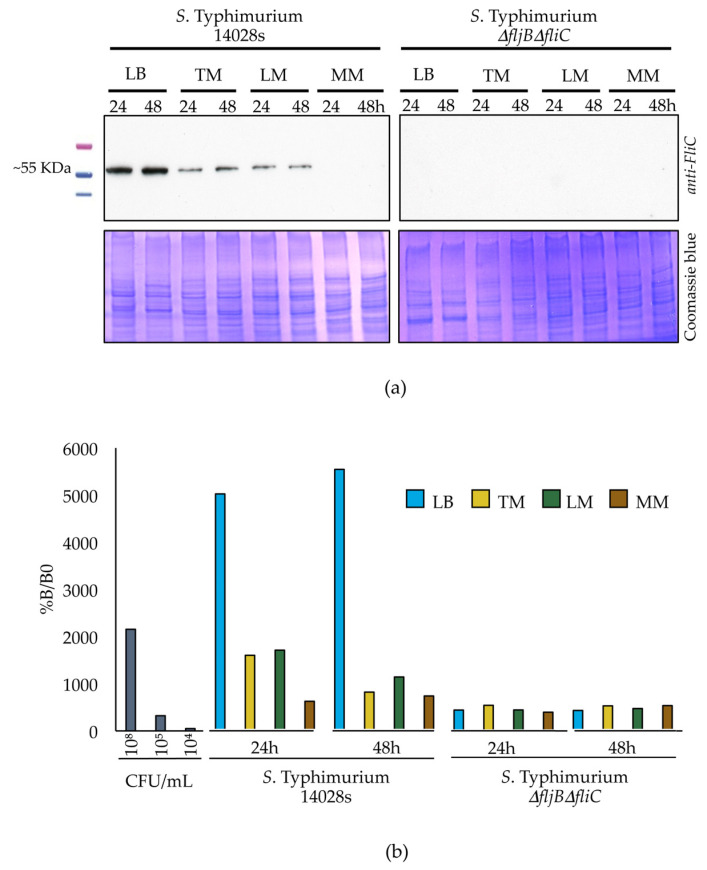
Flagellin synthesis is reduced in plant-based media. Presence of flagellin protein was determined using western blot (**a**) and ELISA (**b**) techniques. Total *Salmonella* proteins were extracted 24 or 48 h after inoculation of *S.* Typhimurium 14028s into LM, TM, MM or LB media. Inoculation with the *S.* Typhimurium *ΔfljBΔfliC* mutant was used as a negative control. Five µg of total protein was separated on SDS–PAGE and blotted on a PVDF membrane prior to probing with a primary anti-*Salmonella* specific flagellin (*anti-FliC*) antibody, followed by probing with secondary anti-mouse antibody coupled to HRP enzyme and exposition (**a**). Alternatively, 96-well plates were coated with proteins isolated as indicated and probed with primary (*anti-FliC*) and secondary anti-mouse antibody coupled to HRP antibodies. The resulting substrate production was assessed 30 min after reaction start using 450 nm and 652 nm wavelengths. As internal control, several dilutions of the wild type strain growing in LB at 24 hpi were used to compare the expression level. The dilutions used were 10^8^, 10^5^, and 10^4^ CFU/mL (**b**).

We further verified the down regulation of flagellin synthesis by analyzing the corresponding protein levels. Both strains, *S*. Typhimurium 14028s and *S*. Typhimurium *ΔfljBΔfliC* double mutant, were inoculated into TM, LM and MM media, as described above. The presence of flagellin was assessed 24 and 48 hpi by both western blot and ELISA assays, using an anti-FliC antibody. Results obtained by these two methods were very similar, detecting flagellin in bacteria cultured in LB medium even after 48 h of incubation (Figure 4a). Flagellin levels were drastically reduced in both TM and LM plant-supplemented media, and were undetectable in control MM medium. As expected, flagellin was not detected in the double *S*. Typhimurium *ΔfljBΔfliC* mutant (Figure 4a). In line with these observations, the results obtained using the more sensitive ELISA technique revealed a similar pattern. Flagellin was present in LB-originated samples 24 and 48 hpi, but was detected at significantly lower levels in both plant-supplemented media, and its presence in MM was below the level of detection, as for the *S*. Typhimurium *ΔfljBΔfliC* mutant (Figure 4b).

### 3.4. Flagellin Is Heterogeneously Expressed in the Plant Environment

In *Salmonella*, expression of *fliC* has been described to display high levels of phenotypic heterogeneity due to molecular noise [41]. Thus, we decided to investigate the changes in *fliC* expression at a single-cell level, both in plant-mimicking media and *in planta*.

**Figure 5:**
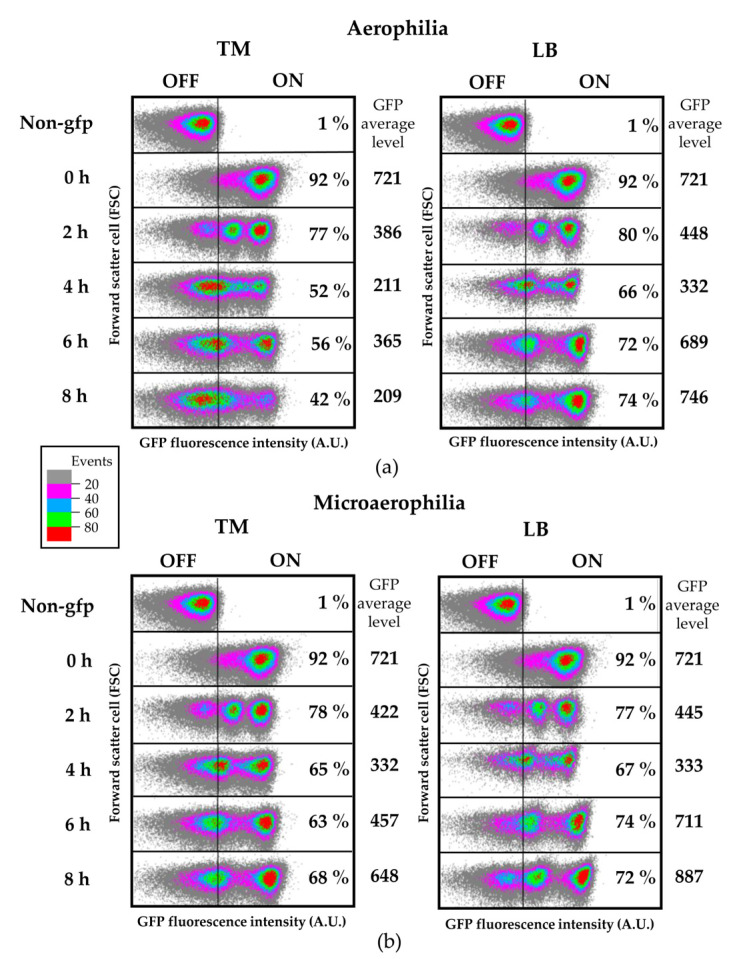
The *fliC* gene is heterogeneously expressed in plant-supplemented media. Dot plots showing time course expression of *fliC::gfp* in tomato-based (TM) and LB media under aerophillic (**a**) and microaerophillic (**b**) conditions. Dot plots represent the GFP fluorescence intensity versus the forward scatter cell or the cell size, both in arbitrary units (A.U.) The OFF subpopulation is defined as the fluorescence level below which 99% of the cells of a non-gfp control strain cultured in the same conditions. Percentages of ON cells are indicated inside the graphs. The average level of GFP intensity for each time and condition is shown to the right of the dot plot.

To this purpose, we generated a chromosome-located transcriptional fusion of *fliC* to the *gfp* reporter gene (*fliC::gfp*) and followed the fluorescence levels at different time points of bacterial proliferation in either LB or TM, using flow cytometry. At the start of the experiment, right after dilution of a stationary LB culture incubated overnight at 37 °C with areation, the vast majority of the cells express *fliC*, as established by comparing fluorescence levels of each individual cell with the levels of fluorescence of a non-gfp *Salmonella* control strain. The OFF subpopulation was hereafter defined as the level of fluorescence at which 99% of the non-gfp control bacteria are included, thus all bacteria displaying higher levels of fluorescence are considered part of the ON subpopulation (Figure 5a). However, even though the initial population mostly expresses *fliC* (92% ON), a heterogeneous pattern emerges as soon as 2 hpi, when an OFF subpopulation can be clearly identified (approximately 20% OFF) for cells grown in either TM or LB. This OFF subpopulation grows up to 58% for 8h in TM, whereas in LB the OFF subpopulation grows up to 26%, therefore a higher proportion of FliC^ON^ bacteria is present in the population growing in LB (Figure 5a). Interestingly, the level of heterogeneity of the population increases during the experiment, to the point of establishing two subpopulations displaying different levels of *fliC* expression, or even three (OFF, ON-low, and ON-high) in the case of LB and TM-grown bacteria at 2 and 4 hpi. In addition we evaluated the potential impact of microaerophillia on *fliC* expression during a similar time-course experiment in both LB and TM media. Microaerophilia mimics a restricted availability of oxygen, as found for example in plant stems. Microaerophilia did not affect expression of *fliC* in LB, however, it did cause a clear increment on the percentage of FliC^ON^ bacteria present in TM at the later time points of the experiment (68% compared to 42% in aerobic conditions at 8 hpi) (Figure 5b).

Finally, it is worth noting that the average level of GFP intensity shown for each time point and condition is indicative of the average of *fliC* expression level in the population. A higher decrease of this value is observed during growth in TM compared to growth in LB, being this difference higher in aerophilic conditions than in microaerophilic.

**Figure 6:**
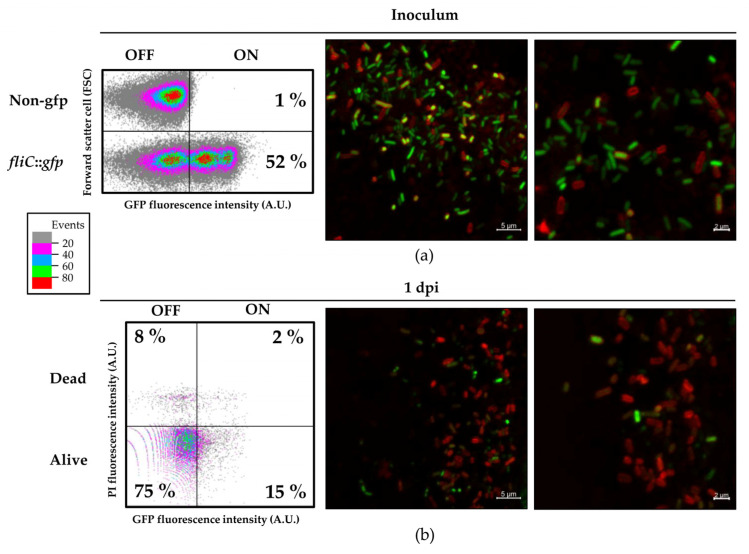
The *fliC* gene is heterogeneously expressed in plants. Dot plot representation of flow cytometry analysis of inoculum (**a**) and apoplast-extracted bacteria (**b**). Dot plots represent the GFP fluorescence intensity versus the forward scatter cell or the cell size, both in arbitrary units (A.U.) (**a**) or the GFP fluorescence intensity versus the propidium iodide (PI) fluorescence intensity, both in arbitrary units (A.U.) (**b**). The OFF subpopulation is defined as the fluorescence level below which 99% of the cells of a non-gfp strain cultured in the same conditions. Apoplast-extracted bacteria were stained with propidium iodide to determine percentage of dead cells. Representative CLSM image of bacterial cells within the inocula (upper panel) and apoplast-extracted bacteria (lower panel) from 1 dpi tomato leaves infiltrated with 5 × 10^8^ CFU/mL. Red corresponds to membrane staining FM4–64. Scale bar corresponds to 5 μm (left panel) and 2 μm (right panel).

Taking advantage of the fluorescent reporter, we further analyzed the impact of the plant environment on the expression of *fliC* by evaluating the GFP expression level by flow cytometry and microscopy, in bacteria directly extracted from the tomato apoplast (Figure 6). Inocula were also analyzed by flow cytometry and CLSM prior to plant infiltration, as a reference. As observed during the time course experiments (24 h), three distinct subpopulations could be clearly identified: OFF, ON-low and ON-high (Figure 6a). Remarkably, the difference in fluorescence intensity between these three subpopulations can be appreciated directly on the bacterial colonies grown in LB used to prepare the inocula, and even as colony sectors (Supplementary Figure S4a). Observations of the *fliC::gfp* strain using CLSM shows representative cells of all three populations in the inoculum (Figure 6a, right panels). One day-post inoculation, bacteria extracted from the tomato leaf apoplast were analyzed by flow cytometry. Prior to the analysis, bacterial cells were stained with propidium iodide (PI) in order to identify dead cells within the apoplastic subpopulation. Apoplast-extracted bacteria were mostly below the level of GFP expression of the control non-gfp bacteria (80–85% FliC^OFF^) (Figure 6b and Supplementary Figure S4b), including 8% of dead bacteria. Only 15–20% of apoplast-extracted bacteria were ON, with levels mostly coinciding with those of the ON-low subpopulation. A similar percentage of dead cells were found in FliC^ON^ and FliC^OFF^ subpopulations, indicating that there is no significant bias towards dead bacteria among the *fliC*-expressing cells. Additional CLSM images confirmed cytometry results, showing examples of bacteria in ON-high state recognizable by very bright GFP fluorescence (Figure 6b).

### 3.5. Recognition of Salmonella in Tomato Is Not Exclusively Based on Flagellin

**Figure 7:**
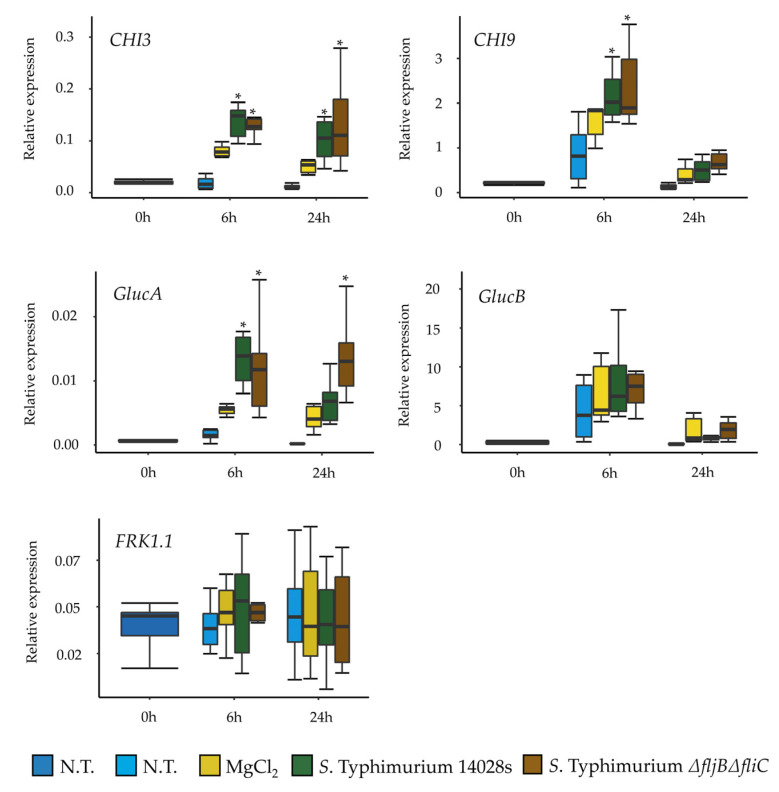
Flagella is not the only PAMP recognized by tomato. Response to inoculation with *S.* Typhimurium 14028s and the double mutant *S.* Typhimurium *ΔfljBΔfliC* was monitored in tomato plants by assessing the transcriptional activation of several *Pathogenesis*-*Related* genes. The expression of *CHI3*, *CHI9*, *GlucA*, *GlucB* and *FRK1.1* was normalized using the expression of tomato *Actin*. The amount of mRNA was assessed in non-treated plants (N.T.) and 6 as well as 24 h (hpi) after challenge with 10 mM MgCl_2_, *S.* Typhimurium 14028s or the *S.* Typhimurium *ΔfljBΔfliC* mutant. The experiment was conducted in six replicates, in each on which three plants per treatment were pooled and used for RNA extraction. Asterisks indicate *p* ≤ 0.05 in Tukey HSD test. For more information about significant differences among treatments, please see Supplementary Table S3.

**Figure 8:**
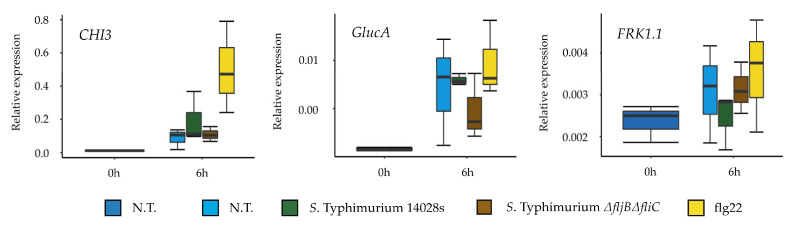
Expression of tomato defense genes is induced upon contact with *Salmonella’s* flagellin. Response to inoculation with *S.* Typhimurium 14028s, the double mutant *S.* Typhimurium *ΔfljBΔfliC* as well as 100 nM flg22 was monitored in tomato plants by assessing the transcriptional activation of *CHI3*, *GlucA*, and *FRK1.1*. The expression was normalized using the expression of tomato *Actin*. The amount of mRNA was assessed also in non-treated plants (N.T.). The experiment was conducted in three replicates, where three plants per treatment were pooled and used for RNA extraction. For information about significant differences among treatments, please see Supplementary Table S3.

The heterogeneous expression of flagellin and the very low percentage of FliC^ON^ bacteria, both ON-low and ON-high bacteria (Figure 6b) in tomato apoplast suggests that the absence of flagellin could be advantageous, perhaps to avoid recognition of this archetypal PAMP by the plant immune system. To get further data on this issue, we monitored the response of tomato plants to spray-inoculation with wild type *S*. Typhimurium 14028s and the *S*. Typhimurium *ΔfljBΔfliC* double mutant. Expression of five defense-associated marker genes, *CHI3*, *CHI9, GlucA*, *GlucB* [27] and *FRK1.1* was analyzed. Expression of all genes (except for *FRK1.1*) was induced 6 h post inoculation, but no significant differences in the plant response were detected after inoculation with either strain (Figure 7). In the case of *CHI9* and *GlucB*, the expression decreased 24 h post inoculation. The lack of differences in response motivated us to verify the response of tomato plants to *Salmonella* flagellin in the absence of bacteria. To this end, we monitored the expression level of *CHI3*, *GlucA* and *FRK1.1* marker genes after treatment with the flg22 peptide, constituted by 22 amino acids corresponding to *S*. Typhimurium flagellin [42]. Strikingly, we could observe an accumulation of *CHI3* transcript and, although to a lower extent, also of *GlucA* and *FRK1.1* (Figure 8). Taken together, these results suggest that even though tomato is able to respond to the presence of *Salmonella* flagellin, *Salmonella* appears to synthesize a reduced amount of flagellin within the plant environment.

## 4. Discussion

During the last decades, there have been many reports on salmonellosis outbreaks associated to fresh produce, leading to public health concerns on the safety of fresh produce and the efficiency of food protection measures during agricultural handling. The most recent outbreak occurred in 14 states in the USA, affected a total of 165 people, 73 of which were hospitalized. The source was associated to cut fruits, including honeydew melons, cantaloupes, pineapples and grapes infected with *S.* Javiana [43].

Several studies have already attempted to comprehend the interaction between *Salmonella* and plant hosts. Flagella’s role in the *Salmonella*-plant interaction has been extensively studied, mostly considering flagellin as a conserved PAMP. Typically, the recognition of PAMPs activates PTI in plants and leads to the onset of several responses, including activation of kinase cascades, production of reactive oxygen species (ROS), activation of the expression of defense genes, stomata closure, and accumulation of defense hormones [14,44,45,46,47]. Nonetheless, several reports have indicated the possible evolvement of divergent flagellin sequences in some bacteria, modifications which would have impact on its recognition and therefore on the interaction between the plant and the bacterium [16,17,18,19]. The N-terminal and C-terminal regions of flagellin are conserved in different bacterial species, while variations take place in the central part. The elicitor activity of flagellin has been attributed to the most conserved domain within the N-terminal part [16,48], which is recognized in plants by the FLS2 receptor [14]. Very intriguing is the fact that the animal ortholog of FLS2 (TLR5) recognizes a different domain [13].

In our previous work [27], results obtained from transcriptomic analysis of *S*. Typhimurium 14028s in tomato-based medium showed that the majority of motility and adhesion related genes were not differentially expressed in this plant-mimicking medium. This led us to speculate that flagella might not be necessary for the survival of *Salmonella* in plants.

Our current work shows that *S*. Typhimurium wild type strain 14028s and the double flagellin mutant *S*. Typhimurium *ΔfljBΔfliC* display the same ability to persist within the inoculated tissue and to colonize non-inoculated areas of tomato plants. Consistent with our results, some studies have reported that *Salmonella* lacking flagellin was able to survive better in alfalfa (*Medicago sativa*) than the respective wild type, suggesting that down-regulation of flagellin synthesis may increase *Salmonella* fitness in plants [22,49]. Likewise, using non-flagellated mutants, flagella were found to be non-essential for attachment of both *S*. Typhimurium and *S*. Senftenberg to tomatoes [20]. Contrarily, in the attachment of *S*. Senftenberg to salad leaves, flagella seemed to play a major role [21]. Furthermore, a recent study showed the importance of flagellum-mediated motility in adhesion of *S*. Typhimurium to *Valerianella locusta* (corn salad) leaves [23]. In addition, *Salmonella* colonization or internalization in *Arabidopsis*, *Medicago sativa* and lettuce was reduced in mutants impaired in motility [50,51]. Noteworthy, it was previously shown that *Salmonella* from infected *Arabidopsis* retain virulence in human cells and mice [52]. This may suggest an adaptive value for the loss of flagella while in contact with plants, thereby gaining more virulence while infecting mammals by not triggering defense mechanisms. Interestingly, previous reports have shown that flagella may play a minor role for endophytic bacteria since endophytes are usually non-motile upon entering plants [49,53,54]. Our results indicate that *Salmonella* inside a tomato plant could adopt a similar lifestyle.

Generally, bacteria express various adhesive structures such as capsule, fimbriae or pili. However, these structures are usually not expressed at the same time as flagellin [55]. For instance, flagella are often involved in reversible attachment that permits individual cells to swim toward suitable biotic or abiotic surfaces. On the other hand, irreversible attachment often involves loss of flagella and synthesis of curli or type 1 fimbriae. Such changes are triggered by different environmental conditions, such as temperature, osmolarity, or pH, which regulate the expression of the flagellar master operon, *flhDC* [47,56]. The assessment of the flagellin expression and synthesis, while in contact with plant-supplemented medium, revealed reduction in both transcript and protein levels of flagellin. This could indicate that *Salmonella* down-regulates the expression of flagella in order to either improve its fitness or to evade plant defense mechanisms associated to the activation of PTI.

Previous reports indicated that *S. enterica* flagellin mutants triggered reduced defense responses in *Arabidopsis* and tomato [57]. This may implicate that tomato, at least partially recognizes *Salmonella’s* flagella as a PAMP. Interestingly, our results showed no differences in the expression of several tomato defense-related genes in plants treated either with the wild type or with the mutant. This raises the question of whether *Salmonella* is generally recognized by tomato Pattern Recognition Receptors (PRRs), and if it does, whether this recognition is due to flagella or to other PAMPs. Another explanation could be attributed to the highly efficient mechanisms of neutralizing ROS that *Salmonella* possesses, which allows it to proliferate inside plants, even when triggering the plant immune response [58,59]. However, our results showed that the expression and synthesis of FliC was higher in LB than in the plant-related media at the population level. Hence, yet another possibility could be that *Salmonella* switches off the production of flagella once in contact with plants in order to evade plant defense system as an evolutionary or adaptive strategy.

Here, we analyzed the *S*. Typhimurium strain expressing a *fliC::gfp* transcriptional fusion to follow *fliC* expression at a single-cell level using both flow cytometry and confocal microscopy. After infiltration of this strain into tomato leaves, we observed significant heterogeneity within the population in regards to the levels of *fliC* expression, detecting FliC^OFF^ and FliC^ON^ bacterial subpopulations. This heterogeneity was also clearly observable using the confocal laser-scanning microscope.

Particularly striking were the big differences in the level of *fliC* expression between individual cells. It was apparent that a small subpopulation of *Salmonella* cells continued to express *fliC* at a very high level. Such heterogeneous expression has been already observed, and even associated to higher virulence in animal hosts. The phenotypic heterogeneity in *Salmonella* flagellar gene expression has been shown to provide a mechanism by which the pathogen maximizes fitness within the mammalian host [60]. Whether such behavior is part of *Salmonella*’s adaptation to colonize plant tissues or plays a role beyond the plant host as part of an adaptation to the eventual progress to an herbivorous host, is a very intriguing question. Further work will be necessary to explore these hypotheses.

Further characterization of additional virulence genes and their expression patterns within the population could define the specificity of interactions between *Salmonella* and crop plants and open doors to new insights of this interaction, leading thereby to the design of informed strategies for enhanced food safety.

## Supplementary Materials

The following are available online at https://www.mdpi.com/2076-2607/8/6/815/s1, **Table S1:** Bacterial strains used in this study [61]. **Table S2:** Oligonucleotides used in this study [62]. **Table S3.** Statistical analysis of results presented in Figure 3, Figure 7 and Figure 8. **Figure S1.** Dipping and infiltration methods used in the translocation assays. **Figure S****2.** Changes in leave appearance caused by *Salmonella* in tomato are not dependent on flagellin. **Figure S3.** Comparison between persistence of *S*. Typhimurium 14028s and the mutant *S*. Typhimurium *ΔfliC* assessed using competitive index assays. **Figure S4.** Populations of *Salmonella* display differences in heterogeneous expression in laboratory rich medium versus plant tissues.
