# Supplementary material for: Salmonella Heterogeneously Expresses Flagellin during Colonization of Plants
Source: Microorganisms. 2020 May 29;8(6):815. doi: 10.3390/microorganisms8060815 (PMC7355505; doi:10.3390/microorganisms8060815)
Supplement: Supplementary file 1 [file microorganisms-08-00815-s001.pdf]

Supplementary Material

Supplementary Tables

Table S1: Bacterial strains used in this study

Strains	Relevant characteristics	Source/reference
S. Typhimurium 14028s	Wt::Rif^r	[26]
S. Typhimurium 14028s	WT- ATCC	[61]
S. Typhimurium *ΔfljBΔfliC*	Rif^r Km^r	Michael Hensel, Osnabrück, Germany
Escherichia coli R751	carrying the helper plasmid R751	[31]
Escherichia coli pSM1890	GFP-tagged	[30]
S. Typhimurium 14028s	GFP-tagged	[27]
S. Typhimurium *ΔfljBΔfliC*	GFP-tagged	This study
S. Typhimurium 14028s	*fliC::gfp*	This study

Table S2: Oligonucleotides used in this study

Oligonucleotide	Sequence (5′–3′)	Reference
CHI3 fw	TGCAGGAACATTCACTGGAG	[62]
CHI3 rev	TAACGTTGTGGCATGATGGT	[62]
CHI9 fw	GAAATTGCTGCTTTCCTTGC	[62]
CHI9 rev	CTCCAATGGCTCTTCCACAT	[62]
GlucA fw	GGTCTCAACCGCGACATATT	[62]
GlucA rev	CACAAGGGCATCGAAAAGAT	[62]
GlucB fw	TCTTGCCCCATTTCAAGTTC	[62]
GlucB rev	TGCACGTGTATCCCTCAAAA	[62]
FRK1.1 fw	CAAAACCCCATCGGCTCTCT	This study
FRK1.1 rev	ACTTCTTTGACACCGCACCA	This study
Actin fw	AGGCACACACAGGTGTTATGGT	[62]
Actin rev	AGCAACTCGAAGCTCATTGT	[62]
fljB fw	TGTTACTATTGGTGGCTTTACTGG	[62]
fljB rev	CAGCAGGCATTGTGGTCTTAG	[62]
fliC fw	AACGACGGTATCTCCATTGC	This study
fliC rev	TACACGGTCGATTTCGTTCA	This study
fimA fw	TGTGCCGTCAGCACTAAATC	This study
fimA rev	TGGTGTTATCTGCCTGACCA	This study
rfaH fw	TCAGCCATTTTGTGCGCTT	Francisco Ramos-Morales, Seville, Spain
rfaH rev	TTCAGGATCGACAACGCCTT	Francisco Ramos-Morales, Seville, Spain

Table S3. Statistical analysis of results presented in Figures 3, 7 and 8
The excel file contains the Tukey HSD test of 95% confident level of the data presented in Figure 3, Figure 6 and Figure 8. The green-labeled cells represent the significant differences among treatments, where $p \leq 0.05$.

Supplementary Figures

Figure S1. Dipping and infiltration methods used in the translocation assays
Four-five-week-old tomato plants growing in greenhouse were either infiltrated (a) or dip inoculated (b) using *Salmonella enterica* serovar Typhimurium strain 14028s (*S.* Typhimurium 14028s) and the double *ΔfljBΔfliC* mutant at either OD_{600nm} = 0.01 (infiltration) or OD_{600nm} = 0.1 (dipping). In order to avoid a cross contamination of the non-inoculated parts, the plants were covered with plastic bags during the inoculation process. The bags were removed carefully after 3hpi to ensure the drying of the inoculated leaves.

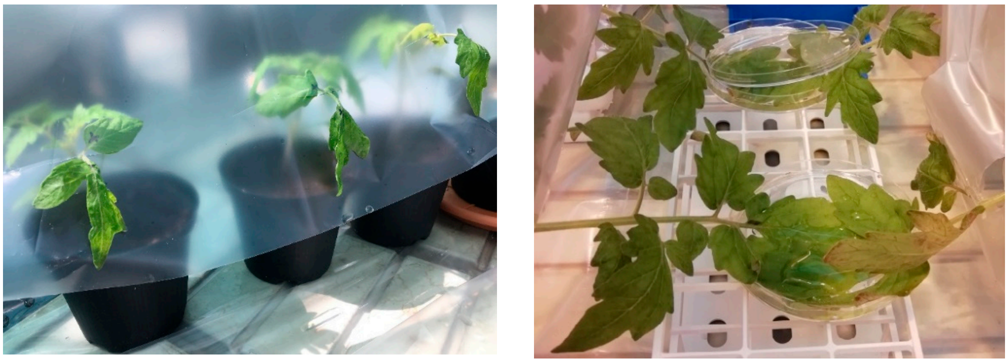

(a) (b)

Figure S2. Changes in leave appearance caused by *Salmonella* in tomato are not dependent on flagellin *Salmonella enterica* serovar Typhimurium strain 14028s (*S*. Typhimurium 14028s) and its double *ΔfljBΔfliC* mutant were infiltrated at OD_{600nm} = 0.01 into tomato leaves. The presence of viable colony forming units (CFUs) was assessed during the following 14 days in cutout leaf discs (Figure 1a). No differences in the phenotypic appearance between leaves infiltrated with the wild type *S.* Typhimurium 14028s and the double *ΔfljBΔfliC* mutant were observed in tomato.

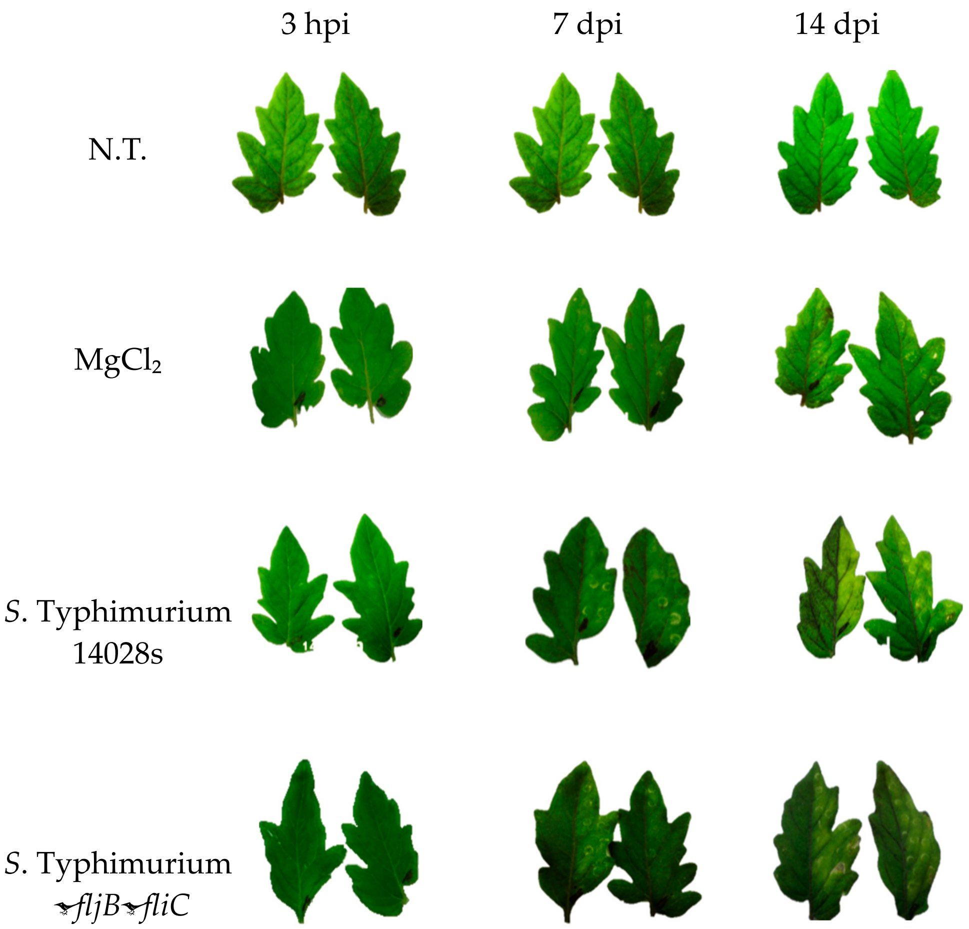

Figure S3. Comparison between persistence of *S*. Typhimurium 14028s and the mutant *S*. Typhimurium *ΔfliC* assessed using competitive index assays
Direct comparison between persistence of *S*. Typhimurium 14028s and the mutant *S*. Typhimurium *ΔfliC* was assessed using competitive index (CI) assays. Both strains were co-inoculated by infiltrating a $5x10^5$ CFU/mL with a 1:1 proportion of these two strains. Bacteria were extracted 4, 7 and 18 dpi from tomato leaves and plated on LB plates for CFU determination. Replica plating was carried out in LB and LB+ kanamycin to differentiate between the two strains. CIs presented show mean values of five replicates. Errors bars represent standard error. Each CI was analyzed using a homoscedastic and 2-tailed Student's *t*-test and the null hypothesis that mean index is not significantly different from 1, $p < 0.05$ were considered significant.

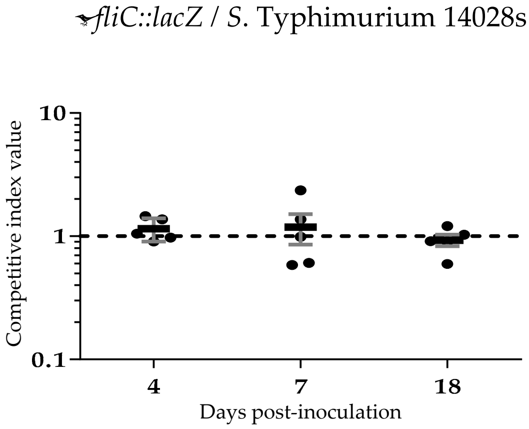

Figure S4. Populations of *Salmonella* display differences in heterogeneous expression in laboratory rich medium *versus* plant tissues
Representative image showing differences in GFP fluorescence between colonies and even between sectors within a single colony (a). Scale bar correspond to 100 µm (left panel) and 20 µm (center and right panels). Replica of experiment shown in Figure 5b without staining for propidium iodide (b). Dot plot graph displaying flow cytometry analysis of inoculum (upper graph) and apoplast-extracted bacteria (lower graph). Dot plots represent the GFP fluorescence intensity *versus* the forward scatter cell or the cell size, both in arbitrary units (A.U.) The OFF subpopulation is defined as the fluorescence level below which 99% of the cells of a non-gfp strain cultured in the same conditions. Percentages of ON cells are indicated. Representative CLSM image of bacterial cells within the inocula (upper panel) and apoplast-extracted bacteria (lower panel) from 1 dpi tomato leaves infiltrated with 10^8 CFU/mL. Red corresponds to membrane staining FM4-64. Scale bar corresponds to 2 µm.

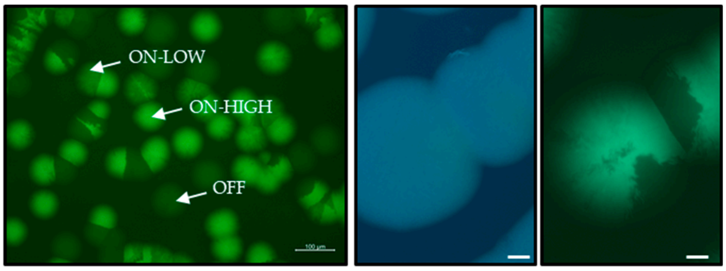

(a)

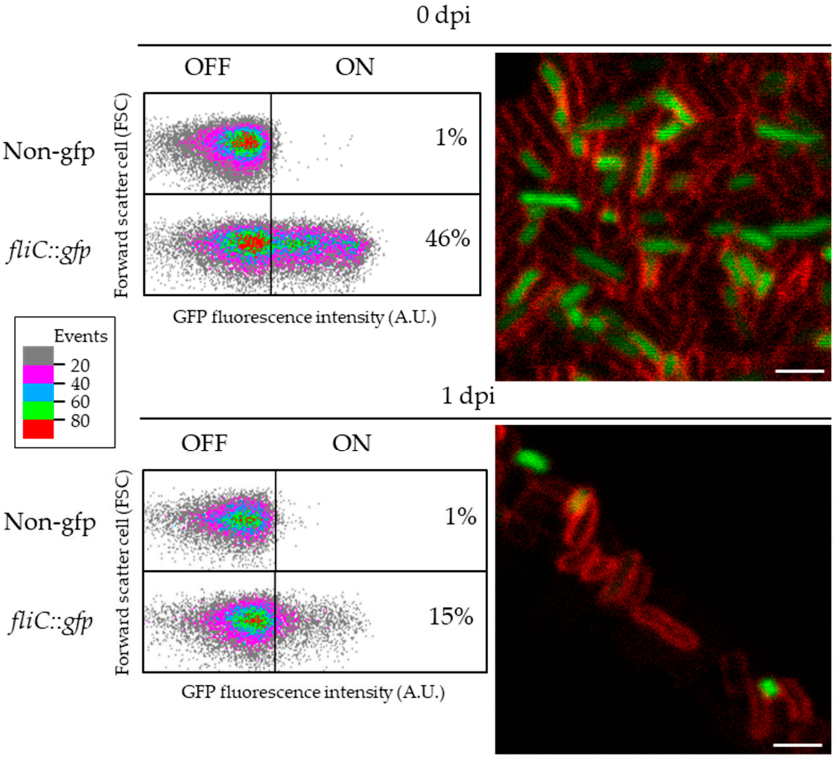

(b)
